# Fucoidan Sulfatases from Marine Bacterium *Wenyingzhuangia fucanilytica* CZ1127^T^

**DOI:** 10.3390/biom8040098

**Published:** 2018-09-21

**Authors:** Artem S. Silchenko, Anton B. Rasin, Anastasiya O. Zueva, Mikhail I. Kusaykin, Tatiana N. Zvyagintseva, Anatoly I. Kalinovsky, Valeriya V. Kurilenko, Svetlana P. Ermakova

**Affiliations:** 1 Laboratory of Enzyme Chemistry, G.B. Elyakov Pacific Institute of Bioorganic Chemistry, Far-Eastern Branch of the Russian Academy of Sciences, 159, Prospect 100-let Vladivostoku, 690022 Vladivostok, Russia; abrus__54@mail.ru (A.B.R.); zstasya95@gmail.com (A.O.Z.); zvyag@piboc.dvo.ru (T.N.Z.); kaaniv@pidoc.dvo.ru (A.I.K.); valerie@piboc.dvo.ru (V.V.K.); 2 School of Natural Sciences, Far-Eastern Federal University, 8, Sukhanova, st., 690091 Vladivostok, Russia

**Keywords:** bacterium *Wenyingzhuangia fucanilytica* CZ1127^T^, sulfated polysaccharides, fucoidan degradation, fucoidan catabolism, sulfated fucooligosaccharides, carbohydrate-sulfatases, fucoidan sulfatases, substrate specificity, 2*O*- and 3*O*-sulfatases

## Abstract

Fucoidans belong to a structurally heterogeneous class of sulfated polysaccharides isolated from brown algae. They have a wide spectrum of biological activities. The complex structures of these polysaccharides hinder structure-activity relationships determination. Fucoidan sulfatases can make useful tools for the determination of the fine chemical structure of fucoidans. In this study, identification and preparation of two recombinant sulfatases able to catalyze the cleavage of sulfate groups from fragments of fucoidan molecules is described for the first time. Two genes of sulfatases *swf1* and *swf4* of the marine bacterium *Wenyingzhuangia fucanilytica* CZ1127^T^ were cloned and the proteins were produced in *Escherichia coli* cells. Sulfatases SWF1 and SWF4 are assigned to S1_17 and S1_25 subfamilies of formylglycine-dependent enzymes of S1 family (SulfAtlas). Some molecular and biochemical characteristics of recombinant fucoidan sulfatases have been studied. Detailed specificity and catalytic features of sulfatases were determined using various sulfated fucooligosaccharides. Structures of products produced by SWF1 and SWF4 were established by nuclear magnetic resonance (NMR) spectroscopy. Based on the obtained data, the enzymes are classified as fucoidan *exo*-2*O*-sulfatase (SWF1) and fucoidan *exo*-3*O*-sulfatase (SWF4). In addition, we demonstrated the sequential action of sulfatases on 2,3-di-*O*-sulfated fucooligosacchrides, which indicates an exolitic degradation pathway of fucoidan by a marine bacterium *W. fucanilytica* CZ1127^T^.

## 1. Introduction

Anionic polysaccharides are indispensable attribute of marine algae which are adaptive compounds that ensure the survival of algae in the marine environment due to their physicochemical properties [1,2]. Fucoidans are representatives of the class of structurally complex sulfated polysaccharides encountered in brown algae. Fucoidans possess a wide spectrum of biological activities depending on their structural features [3]. The main building block of these biopolymers is a sulfated L-fucose residues [3]. In simplest cases L-fucose residues form a polymeric core chain either by α-(1→3)- or alternating α-(1→3)- and α-(1→4)-glycosidic bonds. The remaining hydroxyl groups in fucose residues can be substituted by sulfate groups, acetate groups and fucose residues or other monosaccharides as branches.

Enzymatic machinery that is involved in the catabolism of these polysaccharides is poorly understood. To date, only a few enzymes involved in the depolymerization and deacetylation of fucoidans have been expressed and biochemically characterized [4,5,6,7]. Enzymes that catalyze the further steps of the catabolic process have not been described. It has been shown that the use of highly specific fucoidanases to establish of structure of fucoidans is a very effective approach [5,6,8,9,10]. Despite this, an increase in the number of highly specific enzymes is necessary to improve existing or develop new methods for studying the structures of these complex polysaccharides.

Sulfatases play a key role in the catabolism of various sulfated polysaccharides of marine origin (ulvans, carrageenans, agarans, fucoidans, etc.). Most characterized carbohydrate sulfatases belong to the S1 family and catalyze the cleavage of sulfate groups through the hydrolytic mechanism (SulfAtlas classification) [11]. Members of this family are Cα-formylglycine-dependent (FGly-dependent) enzymes in which one of the cysteine (Cys) or serine (Ser) residues undergoes a post-translational modification and is converted to a Cα-FGly residue [11]. The large variety of sulfated polysaccharide structures implies a large amount of sulfatases with different substrate specificity. Despite advances in processing and annotating of the genomic and metagenomic data of marine microorganisms, the correct functional annotation of carbohydrate sulfatases is still difficult, due to the fact that some sulfatases have not yet been discovered and sulfatases acting on many marine polysaccharides have been poorly characterized [12]. To date, only a few carrageenan sulfatases and agaran sulfatases have been biochemically characterized [12]. There are only fragmentary data about fucoidan sulfatases: several reports of the presence of fucoidan sulfatase activity in some marine bacteria [13,14,15,16,17] and invertebrates [18,19,20]. Substrate specificity is described only for a one semi-purified sulfatase preparation from the marine mollusc *Pecten maximus* [19]. Amino acid sequences, specificities, mode and mechanism of action of fucoidan sulfatases are still unknown.

The goals of this study were to identify and functionally characterize fucoidan sulfatases from fucoidan-degrading marine bacterium *Wenyingzhuangia fucanilytica* CZ1127^T^, carry out a detailed analysis of their amino acid sequences, and establish their detailed substrate specificity for further use as tools for establishing the structures of fucoidan molecules.

## 2. Materials and Methods

### 2.1. Reagents

The strain of marine bacterium *W. fucanilytica* CZ1127^T^ was purchased from the Korean Collection for Type Cultures (KCTC No. 42864) (181 Ipsin-gil, Jeongeupup, Jeollabuk-do, Korea). Sulfated fucooligosaccharides and their mixtures (low molecular weight products (LMP fractions)) were obtained by enzymatic hydrolysis of fucoidans from *Fucus evanescens* and *Sargassum horneri* by recombinant fucoidanases FFA1 and FFA2 from marine bacterium *Formosa algae* KMM3553^T^ as described in [5,6]. Crude fucoidans from *S. horneri* and *F. evanescens* were obtained as described in [6,21].

### 2.2. General Methods

The total carbohydrate amount was determined using the phenol–sulfuric acid method with L-fucose as the standard [22]. The protein concentration was determined by the Bradford method [23] with bovine serum albumin as a standard. Isolation and purification of genomic DNA were performed using the GenElute Bacterial Genomic DNA Kit (Sigma, St. Louis, MO, USA) according to the manufacturer’s protocol.

### 2.3. Identification and Amino Acid Sequences Analysis of Sulfatases

The genome of *W. fucanilytica* CZ1127^T^ (GenBank: GCA_001697185.1) was analysed to reveal the presence of sequences similar to known fucoidanases belonging to 107 family of glycoside hydrolases (CAZy). Sequences of fucoidanases FcnA (GenBank: CAI47003.1), Fda1 (GenBank: AAO00508.1), Fda2 (GenBank: AAO00509.1) and SVI_0379 (GenBank: BAJ00350.1) were used for homology searching in the genome of *W. fucanilytica*. Identification of putative fucoidanases genes were carried out using basic local alignment search tool (BLAST) hits to all proteins in *W. fucanilytica* using GenBank assembly (GenBank: GCA_001697185.1). Four genes of fucoidanases have been identified in the genome of the marine bacterium *W. fucanilytica*. Genes in the immediate vicinity to the fucoidanases genes were manually annotated using the InterProScan server (EMBL-EBI service, Version 5, Hinxton, Cambridgeshire, UK) and CAZy database (Northern Illinois University, db CAN 2 meta server, DeKalb, IL, USA) [24,25]. As a result, the putative fucoidan-utilizing locus and the putative fucoidan sulfatases genes *swf1* (GenBank access: WP_068825883.1) and *swf4* (GenBank access: WP_068828765.1) were identified. The identity of amino acid sequences of the fucoidan sulfatases was performed using the Clustal Omega service (EMBL-EBI service, Hinxton, Cambridgeshire, UK)) [26] and Jalview software (ELIXIR-UK resource, Version 3, Hinxton, Cambridgeshire, UK)) [27]. A signal peptide was predicted using SignalP (Technical University of Denmark, Version 3.0, Lyngby, Denmark) [28]. InterProScan was used for functional domains search [24]. Genome visualization, analysis and reconstruction of fucoidan-utilizing locus were performed using Artemis software (Sanger Institute, Version 15.0.0, Hinxton, Cambridgeshire, UK) [29].

### 2.4. Homology Modeling of SWF1 and SWF4

The Swiss-model server was used to generate homology-based models of SWF1 and SWF4 using protein data bank (PDB) entry 4ug4 and 5g2v as templates, respectively [30]. Energy minimization of resulted structures was performed using yet another scientific artificial reality application (YASARA) (YASARA Bioscience GmbH, Version 17.8.10, Vienna, Austria) [31]. The final models were analysed to check the quality parameters using quality model energy analysis (QMEAN) [32]. Visualization of protein models and structural analysis of *S*-subsites of sulfatases were carried out using PyMol (The PyMOL Molecular Graphics System, Version 1.8, Schrödinger, LLC, New York, NY, USA).

### 2.5. Cloning of Fucoidan Sulfatases SWF1 and SWF4

Constructs were cloned using the restriction-free (RF) cloning strategy [33]. The genomic DNA of *W. fucanilytica* CZ1127^T^ was used as the template for amplification of fucoidanases genes. The constructs for genes of sulfatases *swf1* and *swf4* were designed to harbour a C-terminal his-tag (vector encoded). The genes of sulfatases were amplified in a regular polymerase chain reaction (PCR) with high-fidelity polymerase (New England Biolabs, Ipswich, MA, USA), which produces a primer pair that, once annealed to the 5207–5297 region of pet-22b(+) (Novagene, Madison, WI, USA), is extended in a linear amplification reaction. Primer design and PCR conditions were carried out using service at [34]. The sequence of forward primers for SWF1 was 5′-ATTTTGTTTAACTTTAAGAAGGAGATATACATATGAAATCTCTTAAAAATACCAAGCCTA-3′ and for SWF4 5′-ATTTTGTTTAACTTTAAGAAGGAGATATACATATGAAAGTTGAAAAGAAGCCTAACATTA-3′, reverse primers for SWF1 was 5′-GCTTGTCGACGGAGCTCGAATTTTAATTATTTGTGTTTTTAGGAGCC-3′ and for SWF4 5′-GCTTGTCGACGGAGCTCGAATTTTAGTTTAAAGTTCTTTTAAATAAAGTTTCA-3′, where the underlined sequence is the vector-specific primer and the non-underlined is the gene-specific primer. After RF cloning, parental pet-22b(+) vector was eliminated by treating the reaction mixture with DpnI restriction enzyme (1 µL of 20 U/L for 2 h at 37 °C; New England Biolabs, Ipswich, MA, USA). Then, XL10-Gold ultra-competent cells (Stratagene, San Diego, CA, USA) were transformed by RF products. Colony PCR screening was performed using T7 primers. Positive clones were confirmed by DNA sequencing.

### 2.6. Production of Recombinant Fucoidan Sulfatases SWF1 and SWF4

The protein expression of cloned sulfatases genes was performed using *Escherichia coli* Arctic Express (DE3) strain (Agilent Technologies, Santa Clara, CA, USA). Recombinant strain harboring the pet-22b(+)/*swf1* or pet-22b(+)/*swf4* recombinant plasmid was cultivated on lysogeny broth (LB medium) with ampicillin (100 µg/mL) in shaking incubator at 210 r.p.m. and 37 °C for 16 h. Suspension of culture was inoculated in LB medium (1:100, *v*/*v*) containing ampicillin (final concentration 100 µg/mL) and growth at 210 r.p.m. at 31 °C up to OD_600_ = 0.4–0.6, then temperature was decreased to 18 °C and gene expression was induced by isopropyl *β*-D-1-thiogalactopyranoside IPTG (final concentration 0.8 mM). After induction, transformed bacteria were cultured in a shaking incubator at 230 r.p.m. and 18 °C for 20 h.

### 2.7. Purification of Sulfatases SWF1 and SWF4

All purification steps were performed at 4 °C. Bacterial cells were collected by centrifugation for 30 min at 5500× *g*. The 0.04 M Tris–HCl buffer pH 7.5 (with addition of 0.2 M NaCl and 0.01 M imidazole) was added to 3 g of the bacterial biomass in a 5:1 (*v*/*w*) ratio. This mixture was disrupted by sonication at 20 kHz, five times for 3 min each. The suspension was centrifuged at 12,000× *g* for 40 min to remove the cellular debris. The supernatant (14 mL) was subjected to a HisTrap HP column (5 mL, GE Healthcare) equilibrated with the 0.5 M NaCl, 0.01 M imidazole, 0.01% Triton X100 in 0.04 M Tris–HCl buffer pH 7.5. The proteins were eluted with a linear gradient of imidazole (from 0.02 to 0.3 M) in a buffer (0.04 M Tris–HCl buffer pH 7.5 with 0.5 M NaCl) volume of 50 mL and a flow rate 1 mL/min on Biolodic LP System (Bio-Rad, Hercules, CA, USA). The fractions contained proteins were analyzed by sodium dodecyl sulfate–polyacrylamide gel electrophoresis (SDS-PAGE) for the presence of protein bands with the expected molecular weight. Fractions containing the target proteins were pooled, concentrated on Vivaspin 10 K devices (Sartorius, Epsom, UK) and desalted on a Bio-Scale mini-column with Bio-gel P-6 (10 mL, Bio-Rad, Hercules, CA, USA) equilibrated with 0.06 M Tris-HCl buffer pH 8.0. The resulted fractions were concentrated on Vivaspin 10 K devices (Sartorius AG, Waldbronn, Germany) to a final volume of 1 mL and mixed with 0.5 mL of 80% glycerol. The resulted fractions (1.5 mL) with the target proteins were stored at −20 °C.

### 2.8. Sodium Dodecyl Sulfate Polyacrylamide Gel Electrophoresis of Proteins

The purity and molecular weight of proteins were estimated by SDS–PAGE according to the Laemmli protocol [35]. Electrophoresis was performed in 12% polyacrylamide gels with the addition of detergent—SDS. The Protein Plus molecular weight marker (Bio-Rad, Hercules, CA, USA) with molecular weights of 10–250 kDa was used as standard. Images of gels were obtained using a densitometer GS-800 (BioRad, Hercules, CA, USA). The molecular weights of proteins obtained by PAGE were calculated using the QuantityOne 4.6.7 program (Bio-Rad, Hercules, CA, USA).

### 2.9. Fucoidan Sulfatase Activity Assay

Fucoidan sulfatase activity was monitored by polyacrylamide gel electrophoresis of charged oligosaccharides (C-PAGE) as described in [6]. In brief, the reaction mixture (10 μL) containing 8 μL of enzyme solution (0.01–0.1 mg/mL) in 0.04 M Tris–HCl buffer pH 8.0 with 5 mM CaCl_2_ (buffer A) and 2 μL of sulfated fucooligosaccharides (2.5 mg/mL) or mixture of sulfated oligosaccharides (LMP fractions) (10 mg/mL) solution in buffer A was incubated at 37 °C for 2 h or 24 h. The reaction was stopped by heating at 80 °C for 3 min. The hydrolysis products were mixed with 2.5 μL of loading buffer containing a 20% solution of glycerol in water and 0.02% phenol red. The samples (12 μL) were electrophoresed through a 20% (*w*/*v*) polyacrylamide/bisacrylamide (19:1) gel with 100 mM Tris–borate buffer pH 8.3. The gel was 1 mm thick. Gel staining was performed with a solution containing 0.03% alcian blue 8 GX (Panreac, Barcelona, Spain) in 2% acetic acid for 1 h at room temperature. Washing of the gel was carried out with distilled water until the disappearance of the blue background of the residual dye (but no more than 2 h). Sulfatase activity was detected by the shift or disappearance of bands of sulfated oligosaccharides after enzymatic treatment on electropherogram.

### 2.10. Determination of Substrate Specificity of Sulfatases SWF1 and SWF4

The ability of sulfatases SWF1 and SWF4 to catalyze elimination of sulfate groups from 4-nitrophenyl sulfate, native fucoidans from brown algae *F. evanescens* and *S. horneri*, as well as sulfated fucooligosaccharides have been studied.

The effect of sulfatases on sulfated fucooligosaccharides was evaluated using C-PAGE as described above in Section 2.9.

The ability of sulfatases to catalyze the hydrolysis of sulfate groups from 4-nitrophenyl sulfate was evaluated spectrophotometrically. Reaction mixture (150 μL) containing 0.05 mg/mL sulfatase solution (SWF1 or SWF4) and 4-nitrophenyl sulfate solution 0.5 mg/mL (1.94 mM) in buffer A was icubated for 48 h at 37 °C. The reactions were stopped by adding of 100 μL of 1 M Na_2_CO_3_ solution, and the 4-nitrophenol produced was quantified spectrophotometrically at 410 nm using PowerWave XS plate reader (BioTek, Winooski, VT, USA).

The effect of sulfatases on native fucoidans from *F. evanescens* and *S. horneri* was studied by nuclear magnetic resonance (NMR) spectroscopy. A reaction mixture (2 mL) containing fucoidan (from *F. evanescens* or *S. horneri*, final concentration of 5 mg/mL) and 0.05 mg/mL of sulfatase (SWF1 or SWF4) solution in buffer A was dialyzed at 37 °C for 72 h against buffer A. The reaction products were deproteinised at 85 °C for 10 min and centrifuged for 10 min at 10,000× g. The supernatant was desalted using a Bio-Scale mini-column with Bio-gel P-6 (10 mL, Bio-Rad, Hercules, CA, USA) equilibrated with distilled water. The reaction products were freeze-dried and then analysed by NMR spectroscopy as described in Section 2.15.

### 2.11. Determination of the pH Optimum for Sulfatases SWF1 and SWF4 Activity

The reaction mixture (16 µL), containing 8 µL of enzyme (SWF1 or SWF4) solution (0.01 mg/mL) in buffer A, 5 µL of buffers with various pH values (0.2 M citrate buffers with pH range 4.0–6.5, 0.2 M Tris-HCl buffers with pH values from 6.5 to 8.5 or 0.2 M borate buffer pH 9.0) and 3 µL of sulfated fucooligosaccharides (2.5 mg/mL) in buffer A, was incubated for 2 h at 37 °C. Activity levels were monitored by C-PAGE as described above.

### 2.12. Determination of the Optimal Temperature for Sulfatases SWF1 and SWF4 Activity

The sulfates solutions (8 µL) in buffer A were incubated with 2 µL sulfated fucooligosaccharides (2.5 mg/mL) in buffer A at different temperatures (4, 20, 25, 30, 35, 40, 45, and 50 °C) for 2 h. Enzyme activity was monitored as described above.

### 2.13. Influence of Different Compounds on SWF1 and SWF4 Activity

Enzyme solution (8 µL) was incubated with 2 µL of the appropriate solution (0.1 M BaCl_2_, CaCl_2_, CoCl_2_, CuSO_4_, MgCl_2_, MnCl_2_, NiSO_4_, or 0.5 M EDTA (ethylenediaminetetraacetic acid); 1 M KCl or NaCl) for 10 min at room temperature. After this 2 µL of sulfated fucooligosaccharides (2.5 mg/mL) in 0.04 M Tris-HCl buffer pH 8.0 was added and the mixture incubated for 3 h at 37 °C. Activity levels were monitored by C-PAGE as described above.

### 2.14. Preparation of Reaction Products for Nuclear Magnetic Resonance Analysis

Sulfated fucooligosaccharides **4F2S(4S)** (5 mg) or **4F2,3S(6S)** (3 mg) were dissolved in 0.7 mL of buffer A, and 0.3 mL of SWF1 or SWF4 (0.1 mg/mL) were added. The reaction mixture was incubated at 37 °C for 72 h and then deproteinised by heating at 85 °C for 10 min, precipitate was removed by centrifugation at 10,000× *g* for 10 min. The supernatant containing reaction products was concentrated under vacuum to 0.5 mL and then desalted on Sephadex G-10 column (1.5 × 10 cm). After desalting, the reaction products were freeze-dried and then studied by NMR spectroscopy.

### 2.15. Nuclear Magnetic Resonance Spectroscopy

Nuclear Magnetic Resonance spectra were recorded using Avance DPX-700 NMR spectrometer (Bruker Biospin AG, Fällanden, Switzerland) and Avance DPX-500 NMR spectrometer (Bruker, Hamburg, Germany). 1H, 13C, 1D TOCSY (total correlated spectroscopy) spectra and two-dimensional (2D) spectra (correlation spectroscopy COSY, rotating frame nuclear Overhauser effect spectroscopy ROESY, nuclear Overhauser effect spectroscopy NOESY, heteronuclear single-quantum correlation spectroscopy HSQC, heteronuclear multiple-bond correlation spectroscopy HMBC) were recorded for solutions of poly- and oligosaccharides in D_2_O at 35–40 °C with acetone as the internal standard. The concentration of the samples was 3–10 mg/mL.

## 3. Results

### 3.1. Amino Acid Sequence Analysis of SWF1 and SWF4

Previously, it was shown that marine bacterium *W. fucanilytica* CZ1127^T^ isolated from seawater [36] was able to utilize sulfated fucans extracted from several sources, including sea cucumbers and brown algae [17]. The genome of this marine bacterium was sequenced. Analysis of the genome revealed more than 80 sulfatases genes. Some sulfatases genes, named by us as *swf1* (GenBank access: WP_068825883.1) and *swf4* (GenBank access: WP_068828765.1), are located in close proximity to fucoidanases genes (GH107 CAZy), indicating their putative selectivity to the cleavage of sulfate groups of fucoidans or fucooligosaccharides (Figure S1).

The molecular weight of the product of the *swf1* gene is 55.94 kDa (495 amino acid residue), and *swf4* is 57.08 kDa (496 amino acid residue). The predicted isoelectric points of sulfatases are pH values 8.25 for SWF1 and, 8.61 for SWF4.

The predicted functional domain architecture of sulfatases SWF1 and SWF4 included signal sequences only. The lengths of signal sequences are 24 amino acid residues for both enzymes. Other domains besides catalytic domains were not detected. The identity of amino acid sequences of sulfatases SWF1 and SWF4 among themselves is 27%. The BLAST analysis of amino acid sequences of SWF1 and SWF4 against SulfAtlas database allowed to be assigned to the S1 family, in which SWF1 belongs to subfamily S1_17, and SWF4 to subfamily S1_25. The most similar amino acid sequences of sulfatases were found in genomes of marine bacteria *F. algae* and *Formosa haliotis* (more than 80% identity).

**Figure 1:**
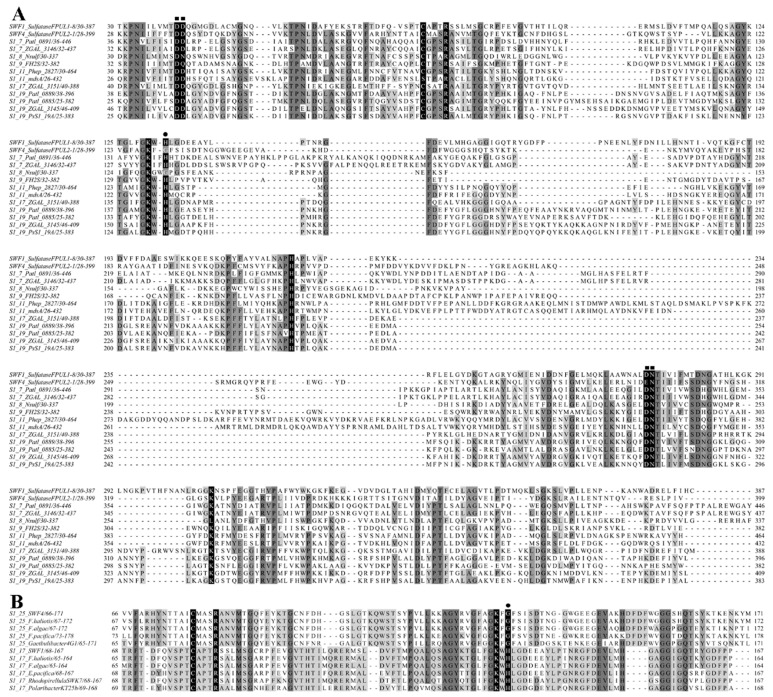
Multiple alignment of sulfatases SWF1 (subfamily S1_17), SWF4 (subfamily S1_25), (**A**) biochemically characterized carbohydrate sulfatases from bacteria (S1_7_Patl_0891—*Pseudoalteramonas atlantica* T6c, *endo*-4*S*-kappa-carrageenan sulfatase (UniProt code: Q15XH1); S1_7_ZGAL_3146—*Zobellia galactanivorans*, *endo*-4*S*-kappa-carrageenan sulfatase (UniProt code: G0L7B4); S1_8_Nsulf—*Pedobacter heparinus* ATCC 13125T, heparin/heparan *N*-sulfamidase (UniProt code: C6Y1N3); S1_9_FH2S—*P. heparinus* ATCC 13125T, heparin/heparan sulfate 2-*O*-sulfatase; S1_11_Phep_2827 *P. heparinus* ATCC 13125T heparin/heparan sulfate 6-*O*-sulfatase (UniProt code: C6Y1N4); S1_11_mdsA—*Prevotella* sp. RS2, mucin-desulfating sulfatase (UniProt code: Q9L5W0); S1_17_ZGAL_3151—*Zobellia galactanivorans*, *exo*-2*S*-alfa-carrageenan sulfatase (UniProt code: G0L7B6); S1_19_Patl_0889—*P. atlantica* T6c, *endo*-4*S*-iota-carrageenan sulfatase (UniProt code: Q15XH3); S1_19_Patl_0885 Q15XG7 *P. atlantica* T6c, *endo*-4*S*-kappa-carrageenan sulfatase (UniProt code: Q15XG7); S1_19_ZGAL_3145—*Z. galactanivorans*, *endo*-4*S*-iota-carrageenan sulfatase (UniProt code: G0L000); S1_19_PsS1_19A *Pseudoalteromonas* sp. PS47 *endo*-4*S*-iota-carrageenan sulfatase (PDB code: 6BIA)) and (**B**) partial sequences of hypothetical sulfatases of subfamilies S1_17 and S1_25 (S1_25_F.haliotis (GeneBank: WP_083191748.1); S1_25_F.algae (WP_069727839.1); S1_25_F.pacifica (WP_044228804.1); S1_25_Gaetbulibacter4G1 (WP_099563503.1); S1_17_F.haliotis (WP_066217792.1); S1_17_F.algae (WP_103190770.1); S1_17_E.pacifica (WP_018474062.1); S1_17_RhodopirellulaSWK7 (WP_009100218.1); S1_17_PolaribacterKT25b (WP_091894582.1). catalytic amino acids of *S*-subsites of sulfatases of the S1 family are indicated by a black background; ●—putative catalytic His and its substitution on Gly in the subfamily S1_25; ■—amino acid residues involved in the binding of metal ions.

Multiple alignments of sulfatases SWF1, SWF4 and biochemically characterized bacterial carbohydrate sulfatases of the S1 family revealed the presence of characteristic conserved amino acid residues. Amino acid pattern CXXXRXXXXXG (Figure 1), characteristic of FGly-dependent enzymes of the S1 family and four conservative amino acids (Asn283, Asp282, Asp40, and Asp41 for SWF1; Asn311, Asp310, Asp38, and Asp39 for SWF4) hypothetically involved in the binding of metal ions were identified in SWF1 and SWF4. Analysis of the amino acid sequence of the obtained enzymes showed that sulfatase SWF1 contains a conserved histidine (His) residue, which in SWF4 was replaced (Figure 1A) to the glycine (Gly) residue (^127^KF**G**^130^). Multiple alignment of the amino acid sequences of sulfatases SWF1 and SWF4 and other sulfatases of subfamilies S1_17 and S1_25 revealed that this substitution is characteristic for the SWF4 homologues in the S1_25 subfamily (Figure 1B).

**Figure 2:**
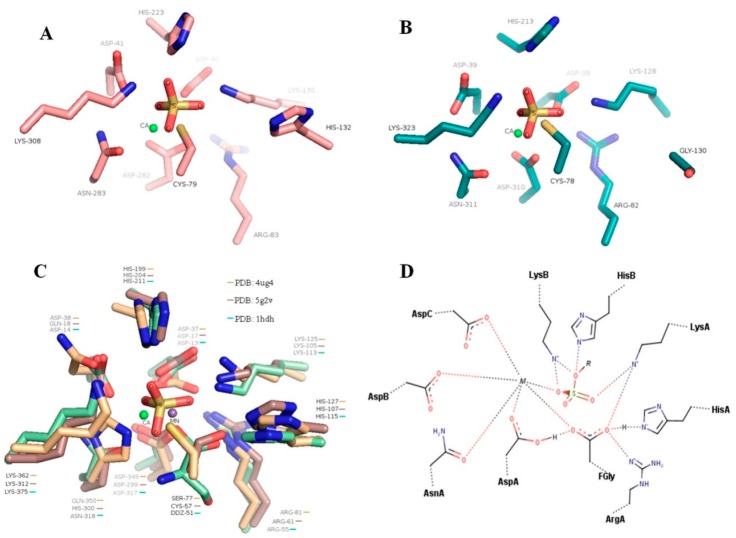
Location of amino acids in *S*-subsites of active sites region of homologous models of sulfatases SWF1 (**A**), SWF4 (**B**) and structural alignment of *S*-subsites of sulfatases from *Bacteroides thetaiotaomicron* BT4656, choline sulfatase from *Sinorhizobium melliloti* and arylsulfatase from *Pseudomonas aeruginosa* (protein data bank (PDB) entries: 5g2v, 4ug4, and 1hdh) (**C**). (**D**) Schematic arrangement of amino acids residues in sulfate-binding *S*-subsites of sulfatases which is proposed in [37]. The proposed function of the amino acid residues is shown in Table 1.

To validate the function of some conserved amino acids identified by the multiple alignments the homology-based structural models of the SWF1 and SWF4 were constructed. According to proposed conventional mechanism, 10 amino acid residues participate in sulfate binding and cleavage [37], forming a highly conserved sulfate-binding site or *S*-subsite (according to subsites nomenclature [38]). Structural alignment of SWF1 and SWF4 with structures of several characterised sulfatases allowed us to identify the features of *S*-subsites organization of putative fucoidan sulfatases (Figure 2A–D). Data on the location of these amino acids and their assumed function are summarized in Figure 2 and Table 1. The *S*-subsite of SWF1 almost completely coincided with the *S*-subsites of known structures of sulfatases S1 family, except some variable metal-binding amino acids, while the *S*-subsite of sulfatase SWF4 is unusual. As we mentioned above, some conserved His in SWF4 is replaced by Gly. According to the proposed conventional mechanism replaced residue of His in SWF4 is canonical HisA (Figure 2D). This residue catalyzes the proton detachments from the geminal hydroxyl group of the FGly-residue and is involved to further desulfation of the intermediate sulfoenzyme complex [37]. This observation is unusual from the point of non-canonical mechanism of desulfating catalysed by SWF4. However, further studies of the SWF4 structure is necessary to make conclusions regarding the mechanism of desulfation catalysed by this enzyme.

### 3.2. Expression and Purification of Fucoidan Sulfatases SWF1 and SWF4

**Figure 3:**
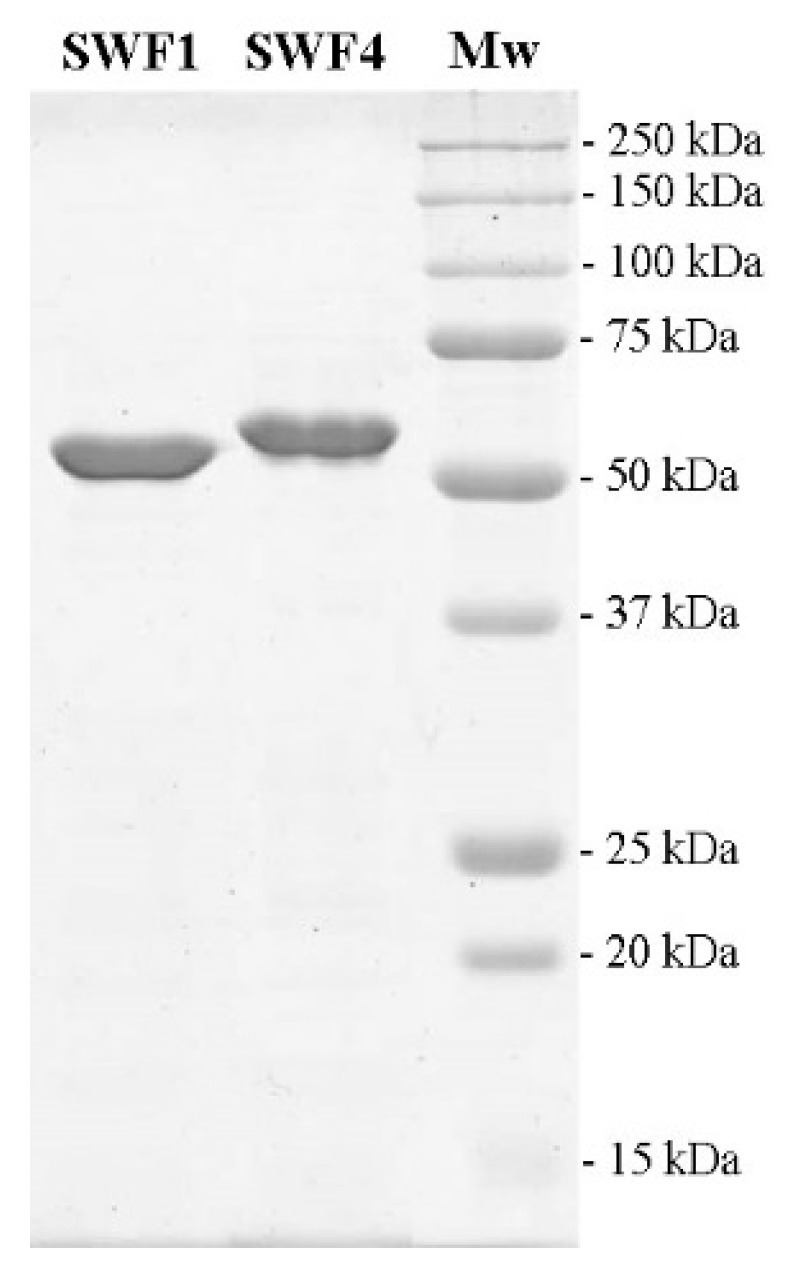
12% Sodium dodecyl sulfate (SDS)-electrophoresis of purified recombinant sulfatases SWF1 and SWF4. MW: molecular weight.

The genes of *swf1* and *swf4* were cloned without the predicted signal sequences. Resulted putative sulfatases gene products of SWF1 (K25-N495) and SWF4 (K25-N496) were produced in *Escherichia coli* Arctic Express strain. The appearance of catalytic activity of sulfatases of S1 family requires a post-translational conversion of Cys or Ser to a catalytic nucleophile FGly. The presence of such a pathway for the post-translational modification of Cys has previously been shown for strains of *Escherichia coli* [39]. The production level of SWF1 and SWF4 were 11 and 8 mg/L of *E. coli* cells in LB media, respectively. The sulfatases were purified by one step purification on Ni-NTA resin. The molecular weights of purified sulfatases according to the SDS-electrophoresis were 55 and 58 kDa for SWF1 and SWF4 respectively, which corresponds to the expected molecular weight of resulted recombinant proteins (Figure 3).

### 3.3. Optimal Conditions for Catalytic Activity of SWF1 and SWF4

Sulfatases SWF1 and SWF4 weakly catalyse the hydrolysis of the artificial substrate 4-nitrophenyl sulfate which make one a poor for studying of their catalytic properties. Therefore, we screened fucoidan sulfatase activity of SWF1 and SWF4 against fucoidan-derived sulfated oligosaccharides. Oligosaccharides from the brown algae *F. evanescens* and *S. horneri* produced by fucoidanases FFA1 and FFA2 with known structures [5,6] were used in this experiment. Polyacrylamide gel electrophoresis (C-PAGE) was used for detection of the activity of sulfatases. The presence of catalytic activity was estimated by changes in the electrophoretic mobility or disappearance of the reaction products on the electropherogram. This approach allowed selecting suitable substrates for studying of catalytic properties of sulfatases. Tetrasaccharide **4F2S(4S)** obtained by the action of fucoidanase FFA2 on fucoidan from *F. evanescens* [5] was used as a substrate for SWF1 and the tetrasaccharide **4F2,3S(6S)** obtained by the action of fucoidanase FFA1 on fucoidan from *S. horneri* [6] was used for SWF4 (detailed discussion described below in Section 3.2).

**Figure 4:**
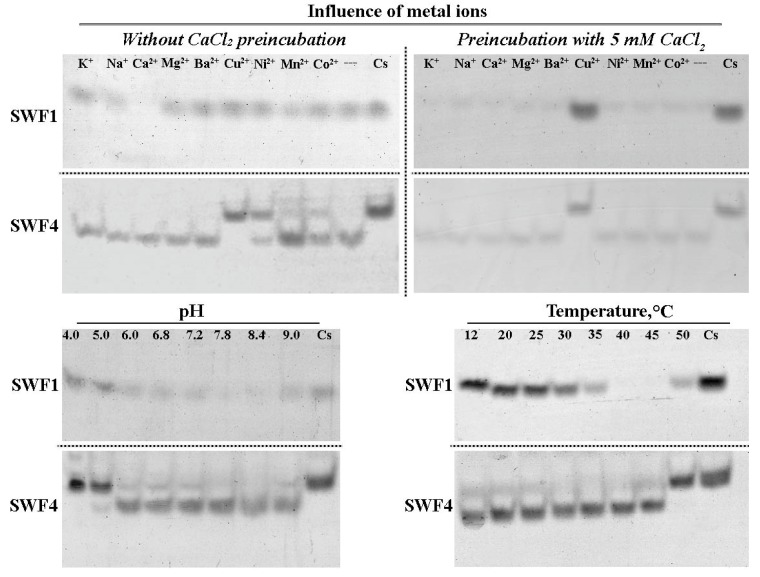
The electropherogram of the action of sulfatases SWF1 and SWF4 on oligosaccharides **4F2,3S(6S)** (for SWF4) and **4F2S(4S)** (for SWF1) at different temperatures; the presence of buffers with different pH values and metal salt solutions; Value of pH and temperatures as well as ions of metals are indicated at top of gels. Cs—oligosaccharides without addition of sulfatases. «---»—incubation mixture without the addition of any metal ions.

It is known that most sulfatases of S1 family are metal-dependent enzymes [37]. The described sulfatases of the S1 family can use either ions Ca^2+^, Mg^2+^ or Mn^2+^ as a cofactor. Therefore, we studied the effect of metal ions on the activity of fucoidan sulfatases. Sulfatase SWF1 exhibited catalytic activity only in the presence of Ca^2+^ ions. The other divalent ions did not able to activate the SWF1 (Figure 4). Thus, fucoidan sulfatase SWF1 is a calcium-dependent enzyme. Sulfatase SWF4 exhibited catalytic activity without addition of any metal ions, and EDTA did not inhibit the catalytic activity of SWF4 (Figure S2). To investigate the inhibitory effect of some divalent ions on sulfatases, they were preincubated with calcium chloride, after which metal ions were added to the incubation mixture. It is shown, that the strong inhibitory effect on both sulfatases possessed only Cu^2+^ ions. Interestingly, the slightly inhibitory effect of Mn^2+^, Ni^2+^, and Co^2+^ on SWF4 was observed only in the absence of Ca^2+^ ions (Figure 4).

The known bacterial carrageenan and agaran sulfatases are active at neutral or slightly alkaline pH values [12]. Both putative fucoidan sulfatases were not an exception and are catalytically active over a wide pH range from 6.0 to 9.0, with an optimum pH from 7.8 to 8.4 (Figure 4). The temperatures optima of studied enzymes differ: for SWF1 it is 40–45 °C, and for SWF4 it is 30–35 °C (Figure 4).

### 3.4. Substrate Specificity and Mode of Action of SWF1 and SWF4

**Figure 5:**
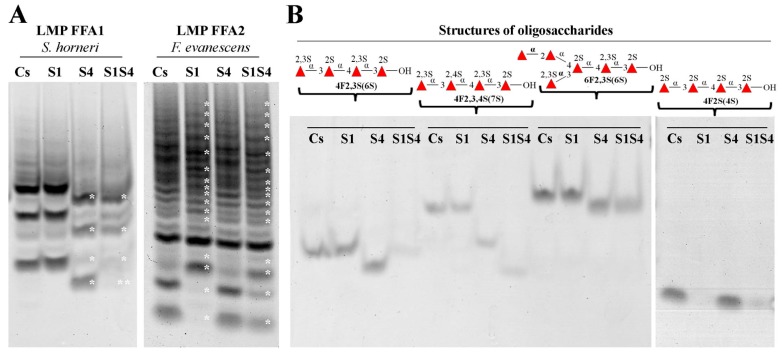
The electropherogram of the action by sulfatases SWF1 (S1), SWF4 (S4) and sulfatases mixture (S1S4) on a mixture of low molecular weight products (LMP) of *S. horneri* and *F. evanescens* fucoidans’ enzymatic hydrolysis by FFA1 and FFA2 (**A**) and oligosaccharides with defined structures (**B**). Structures of oligosaccharides used are schematically represented at the top of gels. *—visible changes under the influence of sulfatases on LMP fractions compared to substrate control (Cs); **—visible changes with the simultaneous action of sulfatases SWF1 and SWF4 (S1S4) compared to the action of SWF4 alone.

As mentioned above both sulfatases very poorly catalysed the hydrolysis of the chromogenic substrate 4-nitrophenyl sulfate. Additionally, SWF1 and SFW4 have no action on native fucoidans from *F. evanescens* or *S. horneri*. We hypothesised that the native substrates of fucoidan sulfatases are the oligosaccharides produced by fucoidanases (GH 107, CAZy). Low molecular weight products of enzymatic hydrolysis and some sulfated fucooligosaccharides were obtained as described earlier and used as substrates [5,6]. These oligosaccharides have a similar glycosidic bonds pattern but differ by the sulfate groups’ position of fucose residues. To specificity investigation we used two mixtures of oligosaccharides (**LMP FFA1** *S. horneri* and **LMP FFA2** *F. evanescens*) and four individual oligosaccharides (**4F2S(4S)**, **4F2,3S(6S)**, **4F2,3,4S(7S)** and **6F2,3S(6S)**) (Figure 5).

The presence of specific activity was evaluated by C-PAGE from the changes of electrophoretic mobility of bands of sulfated oligosaccharides under the action of sulfatases compared to native oligosaccharides (Figure 5).

No visible changes on the electropherogram were observed by the action of SWF1 on the mixture of oligosaccharides **LMP FFA1**, while a shift of bands of sulfated oligosaccharides under the action of the SWF4 on this fraction was detected (Figure 5A). The shift and disappearance of some bands of sulfated oligosaccharides were observed on the electropherogram under the action of SWF1 on the **LMP FFA2** fraction. Sulfatase SWF4 did not act on this fraction, which is confirmed by the absence of visible changes on the electropherogram (Figure 5A). Differences in the actions of sulfatases SWF1 and SWF4 on LMPs fractions is explained by the differences in substrate specificity of sulfatases.

Further we tested an effect of a mixture of SWF1 and SWF4 on the LMP fractions. When mixture of SWF1 and SWF4 were incubated with **LMP FFA1** a further change in the electrophoretic mobility of the oligosaccharides were observed (Figure 5A). These indicate that further desulfation of **LMP FFA1** occurred under simultaneous action of sulfatases SWF1 and SWF4. Deeper desulfation of the **LMP FFA2** fraction under the action of a mixture of sulfatases was not observed (Figure 5A).

The effect of sulfatases SWF1 and SWF4 on distinct oligosaccharides differing by position of sulfate groups and presence/absence of branches were studied (Figure 5B). Sulfatase SWF1 did not catalyse desulfation of oligosaccharides **4F2,3S(6S)**, **4F2,3,4S(7S)** and **6F2,3S(6S)** but desulfate 2*O*-sulfated tetrasaccharide **4F2S(4S)**. On the opposite, the sulfatase SWF4 catalyzed desulfation of oligosaccharides **4F2,3S(6S)**, **4F2,3,4S(7S)** and **6F2,3S(6S)**, but not tetrasaccharide **4F2S(4S)** (Figure 5B).

Incubation of linear sulfated tetrasaccharides **4F2,3S(6S)** and **4F2,3,4S(7S)** with sulfatases mixture led to deeper desulfation compared to the treatment by SWF4 alone. Interestingly, that further desulfation of hexasaccharide **6F2,3S(6S)** (which is the C4-branched analogue of **4F2,3S(6S)**) through sulfatases mixture treatment did not occur. (Figure 5B).

Sulfatases catalyzing the cleavage of sulfate groups from carbohydrate molecules can be distinguished by their mode of action [38]. Like *O*-glycoside hydrolases, sulfatases have an effect on a substrate molecule as either *endo*- or as *exo*-acting enzymes. *Endo*-acting sulfatases cleave sulfate groups located along the chain of poly- or oligosaccharides. A distinctive feature of *exo*-sulfatases is the cleavage of sulfate groups from the non-reducing (NR) or reducing (R) end of substrate molecules.

To determine the mode of action and detailed specificity of sulfatases, the structure of reaction products of sulfatase SWF1 on tetrasaccharide **4F2S(4S)** and sulfatase SWF4 on tetrasaccharide **4F2,3S(6S)** were determined by NMR spectroscopy.

Structure of the tetrasaccharide **4F2F(4S)** from *F. evanescens* was previously determined to be α-L-Fucp(2SO_3_^−^)-1→3-α-L-Fucp(2SO_3_^−^)-1→4-α-L-Fucp(2SO_3_^−^)-1→3-α-L-Fucp(2SO_3_^−^) [5]. The modified tetrasaccharide’s structure of **4F2S(3S)** was identified through its ^1^H, ^13^C and 1D TOCSY spectra, as well as 2D COSY-45, ROESY, HSQC and HMBC spectra. Its chemical shift data are presented in Table 2.

According to these data, the sample’s carbon backbone is α-L-Fucp-1→3-α-L-Fucp-1→4-α-L-Fucp-1→3-α-L-Fucp. The position of sulfate groups in the tetrasaccharide **4F2S(3S)** was deduced by comparing its protons’ and carbons’ chemical shifts with those of α-methyl-L-fucopyranoside (H1/C1 = 5.19/100.5, H2/C2 = 3.76/69.0, H3/C3 = 3.85/70.6, H4/C4 = 3.80/72.9, H5/C5 = 4.19/67.5, H6/C6 = 1.20/16.5). Significant downfield shifts on positions 8, 14 and 20 (from +0.73 to +0.81 ppm for H2, from +5.6 to +7.6 ppm for C2) indicate the presence of sulfate groups [40]. Therefore, the structure of tetrasaccharide **4F2F(3S)** was found to be α-L-Fucp-1→3-α-L-Fucp(2SO_3_^−^)-1→4-α-L-Fucp(2SO_3_^−^)-1→3-α-L-Fucp(2SO_3_^−^).

Structure of the tetrasaccharide **4F2,3S(6S)** from *S. horneri* was established previously to be α-L-Fucp(2,3SO_3_^−^)-1→3-α-L-Fucp(2SO_3_^−^)-1→4-α-L-Fucp(2,3SO_3_^−^)-1→3-α-L-Fucp(2SO_3_^−^) [6]. Due to insufficient quantity of the modified tetrasaccharide **4F2,3S(5S)**, heteronuclear spectroscopy could not be used. However ^1^H spectrum and 2D COSY-45 and ROESY spectra had considerably fine resolutions, allowing us to identify the tetrasaccharide’s structure (Table 3).

From these data, the sample was found to have the carbon backbone α-L-Fucp-1→3-α-L-Fucp-1→4-α-L-Fucp-1→3-α-L-Fucp. Significant downfield shifts for H2 on positions 2, 8, 14, and 20 (from +0.70 to +0.89 ppm) and for H3 on position 15 (+0.91 ppm) indicate the presence of sulfate groups [40]. Thus, the structure of the tetrasaccharide **4F2,3S(5S)** appears to be α-L-Fucp(2SO_3_^−^)-1→3-α-L-Fucp(2SO_3_^−^)-1→4-α-L-Fucp(2,3SO_3_^−^)-1→3-α-L-Fucp(2SO_3_^−^).

**Figure 6:**
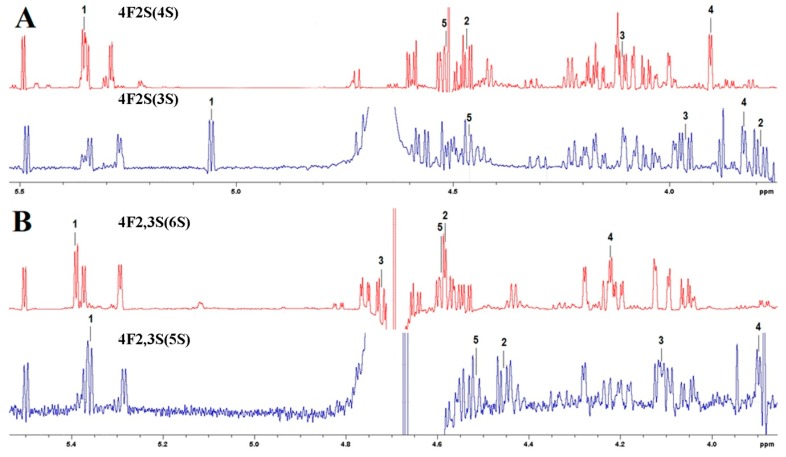
^1^H NMR spectra of tetrasaccharide **4F2S(4S)** before (red) and after (blue) treatment by SWF1 (resulting oligosaccharide **4F2S(3S)**) (**A**) and tetrasaccharide **4F2,3S(6S)** before (red) and after (blue) treatment by SWF4 (resulting oligosaccharide **4F2,3S(5S)**) (**B**); 1–5 are positions of the respective NMR chemical shifts of protons of the tetrasaccharides’ terminal fucose residues.

The effects of sulfatases SWF1 and SWF4 on tetrasaccharides **4F2S(4S)** and **4F2,3S(6S)** respectively are illustrated on Figure 6. SWF1 and SWF4 treatment resulted into prominent upfield shifts (α-desulfation effects) for H2 (Figure 6A) and H3 (Figure 6B) of the first residues from the NR ends. Their adjacent protons showed β-desulfation effects. Thus, SWF4 cleaved off the sulfate group at position 3 of **4F2,3S(6S)**’ first residue from the NR end, while not affecting its sulfate group on position 2, and SWF1 treatment of **4F2S(4S)** cleaved off the sulfate group at position 2 of its corresponding residue. The other residues’ sulfate groups and the glycoside linkages were not affected.

**Figure 7:**
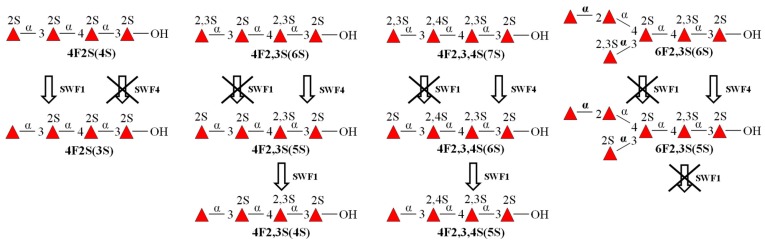
The scheme of sulfatases SWF1 and SWF4’s effects on various sulfated fucooligosaccharides produced by fucoidanases FFA1 and FFA2 from the marine bacterium *Formosa algae*.

Based on the structural data of the desulfation products via sulfatases affecting oligosaccharides, sulfatases SWF1 and SWF4’s expected effects on oligosaccharides used in experiments are summarized in Figure 7.

## 4. Discussion

### 4.1. Identification of Putative Fucoidan Sulfatases in Marine Bacteria Wenyingzhuangia fucanilytica CZ1127^T^

It is known, that genes encoding carbohydrate-active enzymes (CAZYmes) in the genomes of Bacteroidetes phylum bacteria are organized into clusters or polysaccharide utilization loci (PULs) [41]. The PULs have a strict specialization in relation to a polysaccharide type. Function analysis of genes located in close proximity to each other helps to establish the specialization of PULs, as well as to offer the function or specificity of unknown proteins. We used this approach to search for potential fucoidan sulfatases.

The genome of the marine bacterium *W. fucanilytica* CZ1127^T^ was analyzed and a PUL potentially involved in the catabolism of fucose-containing sulfated polysaccharides was identified (Figure S1). Along with fucoidanases (GH107) and fucosidases (GH29 and GH95), this PUL contains genes encoding several sulfatases including *swf1* and *swf4*. We assumed the presence of gene-coding sulfatases in the same locus with fucoidanases (GH 107) to indicate their participation in the catabolism of fucoidans. Similar clusters of genes have been identified in the genomes of marine bacteria *F. algae* and *F. haliotis*. Analysis of the amino acid sequences SWF1 and SWF4 revealed more than 70% homology with sulfatases of these bacteria. In marine bacteria *Flammeovirga pacifica* and *Echinicola pacifica*, clusters of genes containing sulfatase genes homologous to SWF1 and SWF4 (50% and 70% homology, respectively) were detected as well. However, genomes of these bacteria lack homologue genes of fucoidanase GH107. Supposedly, these bacteria are able to catabolize the fucose-containing sulfated polysaccharides but use somewhat different enzyme machineries or are part of a community of microorganisms involved in assimilation of these polysaccharides.

### 4.2. Analysis of Amino Acid Sequences of Putative Fucoidan Sulfatases SWF1 and SWF4

Despite the fact that sulfatases SWF1 and SWF4 perform a similar function, namely the elimination of sulfate groups from a fucose residue, their identity relative to each other is a low 27%. BLAST searches of sulfatases SWF1 and SWF4 in the SulfAtlas database showed that they belong to subfamilies S1_17 (for SWF1) and S1_25 (for SWF4) of the S1 sulfatase family. Representatives of the family S1 are formylglycine-dependent sulfatases (FGly-sulfatases) cleaving a monoester sulfate from the substrate through the hydrolytic mechanism [11]. To date, there is no information about the substrate specificity or crystalline structures of sulfatases belonging to subfamilies S1_25. Thus, sulfatase SWF4 is the first biochemically characterized representative of this subfamily.

To date, the S1_17 subfamily includes biochemically validated sulfatase ZGAL_3151 from the marine bacterium *Zobellia galactanivorans* with 2*O*-sulphatase activity towards alpha-carrageenan fragments [42]. From a chemical point of view, the specificity of SWF1 (fucoidan exo-2*O*-sulfatase) belonging to the same subfamily is similar to ZGAL_3151, since fucose is 6-deoxy-galactose. Thus, at the moment, the subfamily S1_17 includes two biochemically characterized enzymes ZGAL_3151 and SWF1 with 2*O*-sulfatase activity against carbohydrate residues in the galacto-configuration.

### 4.3. Conservativeness of Sulfate-Binding S-Subsites of SWF1 and SWF4

Despite great progress in the study of structures of S1 family sulfatases, the question about the mechanism of sulfate groups’ removal from substrate molecules is still debated [43]. Through template-based structure modeling we constructed homology models of sulfatases SWF1 and SWF4. Structural alignment of these models with well-studied structures of sulfatases made it possible to determine the function of some conservative amino acids in sulfate binding *S*-subsites.

Most structures of sulfatases have a highly conserved sulfate-binding *S*-subsite, formed by 10 polar amino acid residues and a divalent metal cation. The *S*-subsite of sulfatase SWF1 was similar to most of studied S1 family sulfatases. The exception was a metal-binding region with amino acid residues, which vary for some sulfatases, depending on the preference for one or another ion cofactor (Figure 2, Table 1). Interestingly, one of the ten key amino acid residues in *S*-subsite of SWF4, namely HisA, was absent. Similar evidence was observed for the homologues of SWF4 in S1_25 subfamily, which can be a distinguishing feature of this subfamily’s 3*O*-sulfatases. An in silico structural study of *N*-sulfamidase from *Pedobacter heparinus* (*Flavobacterium heparinum*) bacterium by Myette et al. [44] showed HisA to be absent in the active site region of this enzyme. Therefore, the authors proposed a new mechanism of the N–S bound hydrolysis without HisA residue’s participation. In the case of SWF4, this fact requires further structural studies. However, the data obtained indicate the ambiguous role of HisA and probably require further improvement of our knowledge of mechanisms for O–S bound hydrolysis by sulfatases of S1 family. 

### 4.4. Role of a Calcium Cation in Enzyme Activities of SWF1 and SWF4

In most cases the correct orientation and accommodation of the substrate molecule’s sulfate group and FGly residue in active site are required for desulfation process [37]. This is usually achieved via the presence of metal ion in the sulfate-binding *S*-subsite of sulfatases. Together amino acids AsnA, AspA, AspB, and AsnC form a site for metal ions’ binding (Figure 2). These amino acids are also found in amino acid sequences of SWF1 and SWF4 (Table 1). As expected, sulfatase SWF1 is catalytically active only in the presence of Ca^2+^ ions. Interestingly, despite the existence of metal binding site, presence of metal ions in the reaction mixture was not essential for catalytic activity of sulfatase SWF4. Moreover, various experiments with high concentrations of EDTA (up to 50 mM) showed no inhibition of sulfatase activity (Figure S2). However, the presence of catalytic activity in the SWF4 without an addition of divalent cations is not a unique case. Similar results were observed for heparin/heparan sulfate sulfatases [45,46] and N-sulfamidase from *P. heparinus* (*F. heparinum*) [44]. Those enzymes showed catalytic activity in the absence of metal ions in the reaction mixture, however, with the addition of calcium ions the reaction rate increased significantly. Some authors suggested this effect to be associated with the very dense location of the metal ion in the metal-binding pocket of sulfatases, or the inability of chelating agents to interact with this metal ion [45,47].

### 4.5. Substrate Specificity of Sulfatases SWF1 and SWF4

Sulfatases SWF1 and SWF4 showed very low arylsulfatase activity, which indicates their high selectivity with respect to the substrate nature. Thus, an artificial substrate 4-nitrophenyl sulfate often used for search or detection of sulfatase activity may not be effective for reliable detection of such enzymes.

To establish the substrate specificity of sulfatases SWF1 and SWF4, we used a set of sulfated fucooligosaccharides with different structures (sulfation patterns). These sulfated oligosaccharides are the products of biochemically characterized fucoidanases FFA1 and FFA2 from bacterium *F. algae* KMM3553^T^ affecting different fucoidans. The obtained results showed sulfatase SWF1 to catalyze the cleavage of sulfate groups off oligosaccharides sulfated at C2 position of fucose residues only. Structural analysis of the product of sulfatase SWF1’s action on 2*O*-sulfated tetrasaccharide **4F2S(4S)** revealed the cleavage to occur from the NR end. Thus, sulfatase SWF1 is an *exo*-acting enzyme. It is important that sulfatase does not cleave sulfate groups from the 2,3-di-*O*-sulfated oligosaccharides. Thus, the presence of a sulfate group at C3 of terminal fucose residue is a restriction for the action of this enzyme. In contrast to SWF1, sulfatase SWF4 catalyzed the cleavage of sulfate groups from 2,3-di-*O*-sulfated oligosaccharides only. Structural analysis of enzyme action products revealed SWF4 to have a similar mode of action, but to cleave sulfate groups from C3 position of terminal fucose residues. Hence, sulfatase SWF1 can be classified as sulfated fucan *exo*-2*O*-sulfatase and SWF4 as sulfated fucan *exo*-3*O*-sulfatase.

Nuclear Magnetic Resonance spectra analysis of reaction products of sulfatases with native fucoidans did not reveal structural changes. However, the NMR-spectroscopy methods do not have sensitivity sufficient to detect changes in structures of such enzymatic products, since only one sulfate group per polymeric molecule of fucoidan has been removed. Supposedly, sulfatases SWF1 and SWF4 are able to remove sulfate groups not only from oligo-, but also from polysaccharides. The effect of SWF1 sulfatase on almost the all fucoidan fragments in the LMPs fractions regardless of the degree of polymerization of such fragments counts in this statement’s favor (Figure 5A).

We have shown the possibilities of the sequential action of sulfatases on linear and branched sulfated fucooligosaccharides. As noted above, the presence of a sulfate group at C3 on the NR end of the fucooligosaccharides makes the sulfate group at C2 unavailable for SWF1. However, with the simultaneous action of SWF1 and SWF4 on 2,3-di-*O*-sulfated oligosaccharides deeper desulfation occurs. This can be explained by the sequential action of sulfatases on the substrate molecule. At first, sulfatase SWF4 cleaves a sulfate group off C3 position from NR end, making the newly formed oligosaccharide a substrate for SWF1, which cleaves the remaining sulfate off terminal fucose’s C2. Final products of the simultaneous action by these *exo*-sulfatases are partially sulfated oligosaccharides with non-sulfated fucose residues at the NR ends. The results obtained suggest a putative pathway for the flavobacterial degradation of fucoidan. Apparently, a polymeric chain of fucoidan gets cleaved into short fragments by fucoidanases GH107, which then undergo several cycles of sequential action by fucoidan *exo*-sulfatases and 1,3/1,4-fucosidases GH29, resulting into an L-fucose monomer and a sulfate.

It is worth noting that the sequential action of sulfatases SWF1 and SWF4 was observed only for linear oligosaccharides. Specifically, while sulfatase SWF4 did catalyzed the cleavage of the sulfate group from the branched hexasaccharide **6F2,3S(6S)**, the subsequent cleavage of the remaining sulfate group off **6F2,3S(5S)**’s C2 by sulfatase SWF1 did not occur (Figure 5). Meanwhile, the presence of a sulfate group at the same position (С4 of fucose residue following the NR end) in tetrasaccharide **4F2,3,4S(6S)** does not interfere with the action of SWF1. It is evident that a bulky substituent (exclusively a branch) at fucose residue following the NR end in the oligosaccharides interferes with its proper accommodation in the glycone-binding site of SWF1.

The obtained results describe the detailed substrate specificity of fucoidan sulfatases, opening new prospects for study of fucoidans’ detailed structures, in particular, the possibility for sequencing of some fucoidan fragments.

## 5. Conclusions

Genes of two fucoidan sulfatases *swf1* and *swf4* from the marine bacterium *Wenyingzhuangia fucanilytica* CZ1127^T^ were identified for the first time. Amino acid sequence analysis revealed that SWF1 and SWF4 are members of S1_17 and S1_25 subfamilies of S1 sulfatase family (SulfAtlas). Detailed substrate specificity was studied using a set of sulfated fucooligosaccharides, produced by recombinant fucoidanases FFA1 and FFA2. Both sulfatases are *exo*-type enzymes, but have different selectivities towards the location of sulfate groups in fucose residues. Sulfatase SWF1 catalyzes the elimination of sulfate groups from C2 positions of terminal fucose residues in sulfated fucooligosaccharides. This reaction does not proceed if an adjacent sulfate group is present at the C3 position in 2,3-di-*O*-sulfated oligosaccharides or a disaccharide branch is present at C4 of the fucose residue following the NR end. Sulfatase SWF4 catalyzes desulfation of 2,3-di-*O*-sulfated fucooligosaccharides by cleaving off sulfate groups from С3 positions of NR end fucose residues. The cooperative action of SWF1 and SWF4 on fucoidan fragments was demonstrated. The sequential action of these two enzymes on 2,3-di-*O*-sulfated fucooligosaccharides leads to the formation of derivatives with non-sulfated fucose residues at NR ends. The resulted derivative, as we assume, is a necessary step for subsequent fucoidan degradation by fucosidases of marine bacterium *W. fucanilytica* CZ1127^T^.

## Figures and Tables

**Table 1:** Structure-based comparison of sulfate-binding *S*-subsites amino acid residues for several known sulfatases structures and fucoidan sulfatases SWF1 and SWF4.

Residue *	PDB: 5g2v	PDB: 4ug4	PDB: 1hdh	SWF1	SWF4	Proposed Function
***FGly/(modified residue)***	Ser 77	Cys 57	Cys 51	Cys 79	Cys 78	Catalytic nucleophile. Formation of sulfoenzyme intermediate complex.
***M***	Ca^2+^	Mn^2+^	Ca^2^	Ca^2^	Ca^2^	Coordination and stabilization of sulfate group of substrate.
***AsnA***	Gln 350	His 300	Asn 318	Asn 283	Asn 311	Coordination of metal ion.
***AspA***	Asp 349	Asp 299	Asp 317	Asp 282	Asp 310	Coordination of metal ion and activation of FGly.
***AspB***	Asp 37	Asp 17	Asp 13	Asp 40	Asp 38	Coordination of metal ion.
***AspC***	Asp 38	Gln 18	Asp14	Asp 41	Asp 39	Coordination of metal ion.
***LysA***	Lys 125	Lys 105	Lys 113	Lys 130	Lys 128	Sulfate binding and stabilisation of FGly.
***LysB***	Lys 362	Lys 312	Lys 375	Lys 308	Lys 323	Sulfate binding and protonation of ester group of substrate.
***ArgA***	Arg 81	Arg 61	Arg 55	Arg 83	Arg 82	Stabilization of FGly.
***HisA***	His 127	His 107	His 115	His 132	(Gly 130)	Deprotonation of FGly, elimination of sulfoenzyme intermediate complex.
***HisB***	His 199	His 204	His 211	His 223	His 213	Sulfate binding and protonation of ester group of substrate.

* Name of residues and proposed function were generated according to scheme of proposed mechanism for sulfatases (Figure 2) and in [37]; parentheses indicate the amino acids located in the same place in homology model of SWF4 but proposed function is unlikely. Protein data bank (PDB) entries of 5g2v, 4ug4, and 1hdh correspond to sulfatase from *B*. *thetaiotaomicron* BT4656, choline sulfatase from *S*. *melliloti* and arylsulfatase from *P*. *aeruginosa*, respectively. Abbreviations of Arg, Asn, Asp, Cys, Gln, His, Lys and Ser correspond to amino acid residues of arginine, asparagine, aspartic acid, cysteine, glutamine, histidine, lysine and serine, respectively. FGly—Cα-formylglycine residue. M—metal cofactors.

**Table 2:** Nuclear Magnetic Resonance spectroscopic data (500 Hz, D_2_O) for tetrasaccharide **4F2S(3S)** (the product of action of SWF1 on **4F2S(4S)**).

Position	δ_C_, Type	δ_H_ (*J* in Hz)	ROESY	HMBC^α^
1	96.8, CH	5.06, d (3.9)	2, 9, 10	3, 5, 9
2	69.4, CH	3.79, dd (9.9, 3.8)	1	3
3	70.7, CH	3.96, dd (10.3, 3.4)	4, 5	1, 2, 4
4	73.3, CH	3.83, d (3.4)	3, 5, 6	3, 5
5	68.1, CH	4.46, m	3, 4, 6	1, 4, 6
6	16.5, CH_3_	1.21, d (6.6)	4, 5	5
7	100.3, CH	5.27, d (3.3)	8, 16, 18	9, 11, 16
8	74.6, CH	4.57, dd (10.3, 3.6)	7	9
9	72.9, CH	4.18, dd (10.8, 3.1)	1, 10, 11	1, 7, 8, 10
10	70.1, CH	4.11, d (2.2)	1, 9, 11, 12	9, 11
11	68.6, CH	4.44, m	9, 10, 12	7, 10, 12
12	16.5, CH_3_	1.24, d (6.6)	10, 11	11
13	95.4, CH	5.34, d (3.9)	14, 21, 22	15, 17, 21
14	76.6, CH	4.49, dd (10.5, 3.8)	13	15, 16
15	68.6, CH	4.16, dd (10.9, 2.9)	16, 17	13, 14, 16
16	83.7, CH	3.99, d (2.6)	7, 15, 17, 18	7, 14, 15, 16
17	68.9, CH	4.52, m	15, 16, 18	13, 17, 18
18	16.8, CH_3_	1.38, d (6.8)	7, 16, 17	17
19	91.7, CH	5.48, d (3.9)	20	21, 23
20	74.7, CH	4.51, dd (10.2, 3.2)	19	21
21	73.9, CH	4.05, dd (9.9, 3.1)	13, 23	13, 19, 20
22	69.9, CH	4.08, d (3.2)	13, 23, 24	23
23	67.2, CH	4.23, q (13.2, 6.6)	21, 22, 24	19, 22, 24
24	16.7, CH_3_	1.24, d (6.6)	22, 23	23

ROESY—rotating frame nuclear Overhauser effect spectroscopy; HMBC—heteronuclear multiple-bond correlation spectroscopy; **δ_C_**—chemical shift of carbon; **δ_H_**—chemical shift of proton; d—doublet; dd—double doublet; q—quartet; m—multiplet.

**Table 3:** NMR spectroscopic data (500 Hz, D_2_O) for tetrasaccharide **4F2,3S(5S)** (the product of action of SWF4 on **4F2,3S(6S)**).

Position	δ_H_ (*J* in Hz)	ROESY
1	5.36, d (4.6)	2, 9, 10
2	4.46, dd (9.5, 4.1)	1
3	4.11, m	5
4	3.90, d (4.2)	5
5	4.51, m	3, 4
6	1.23, d (6.0)	
7	5.28, d (3.7)	8, 16, 18
8	4.57, m	7
9	4.19, dd (10.4, 3.2)	11
10	4.11, m	11
11	4.43, m	9, 10
12	1.29, d (6.5)	
13	5.37, d (4.4)	21, 22
14	4.65, m	
15	4.76, dd (11.2, 2.8)	16, 17
16	4.28, d (2.9)	7, 15, 17
17	4.55, m	15, 16
18	1.40, d (6.8)	7
19	5.50, d (3.9)	20
20	4.54, m	19
21	4.06, dd (10.2, 3.2)	13, 23
22	4.09, d (4.8)	13, 23
23	4.23, m	21, 22
24	1.24, d (6.4)	

## Supplementary Materials

The following are available online at http://www.mdpi.com/2218-273X/8/4/98/s1, Figure S1: Schematic representation of putative fucoidan-utilization locus of *Wenyingzhuangia fucanilytica* CZ1127^T^, Figure S2: Influence of different concentrations of EDTA on fucoidan sulfatase activity of SWF1 and SWF4.
