# Supplementary material for: Fucoidan Sulfatases from Marine Bacterium Wenyingzhuangia fucanilytica CZ1127T
Source: Biomolecules. 2018 Sep 21;8(4):98. doi: 10.3390/biom8040098 (PMC6315715; doi:10.3390/biom8040098)
Supplement: Supplementary file 1 [file biomolecules-08-00098-s001.pdf]

Supplementary materials

Fucoidan Sulfatases from Marine Bacterium *Wenyingzhuangia fucanilytica* CZ1127^T

Artem S. Silchenko[1,*], Anton B. Rasin[1], Anastasiya O. Zueva[1,2], Mikhail I. Kusaykin[1,*], Tatiana N. Zvyagintseva[1], Anatoly I. Kalinovsky[1], Valeriya V. Kurilenko[1], Svetlana P. Ermakova[1,*]

[1] Laboratory of Enzyme Chemistry, G.B. Elyakov Pacific Institute of Bioorganic Chemistry, Far-Eastern Branch of the Russian Academy of Sciences, 690022, Vladivostok, 159, Prospect 100-let Vladivostoku, Russia

[2] School of Natural Sciences, Far-Eastern Federal University, Vladivostok, 690022, 8, Sukhanova, st., Russia

* Correspondence: ASS, artem.silchencko@yandex.ru, Tel.: +7(423)231-07-05; MIK, mik@piboc.dvo.ru, Tel.: +7(423)231-07-05; SPE, ermakova@piboc.dvo.ru, Tel.: +7(423)231-07-05

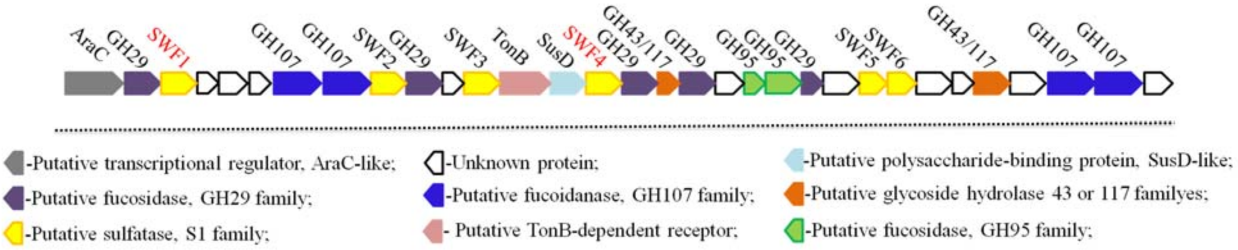

Figure S1. Schematic representation of the putative fucoidan-utilization locus of *Wenyingzhuangia fucanilytica* CZ1127^T (GenBank assembly accession: GCA_001697185.1). Genes of sulfatases *swf1* (GenBank access: WP_068825883.1) and *swf4* (GenBank access: WP_068828765.1) are indicated in red.

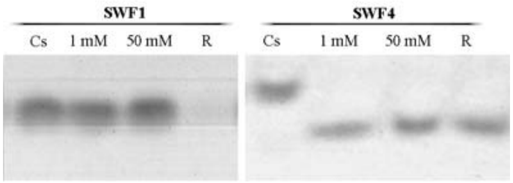

Figure S2. Influence of different concentrations of ethylenediaminetetraacetic acid (EDTA) solutions on fucoidan sulfatase activity of SWF1 and SWF4. Concentrations of EDTA are indicated at the top of the gels. Cs—oligosaccharide control; R—standard enzymatic reaction of SWF1 or SWF4 without addition of EDTA.
